# Influence of Nisin-Biogel at Subinhibitory Concentrations on Virulence Expression in *Staphylococcus aureus* Isolates from Diabetic Foot Infections

**DOI:** 10.3390/antibiotics10121501

**Published:** 2021-12-07

**Authors:** Carolina Jesus, Rui Soares, Eva Cunha, Miguel Grilo, Luís Tavares, Manuela Oliveira

**Affiliations:** CIISA—Centro de Investigação Interdisciplinar em Sanidade Animal, Faculdade de Medicina Veterinária, Universidade de Lisboa, Avenida da Universidade Técnica, 1300-477 Lisboa, Portugal; carolinaojesus@gmail.com (C.J.); rmsoares@fmv.ulisboa.pt (R.S.); evacunha@fmv.ulisboa.pt (E.C.); miguelgrilo@fmv.ulisboa.pt (M.G.); ltavares@fmv.ulisboa.pt (L.T.)

**Keywords:** diabetic foot infections, *Staphylococcus aureus*, subinhibitory concentrations, virulence-related genes, biofilm

## Abstract

A new approach to diabetic foot infections (DFIs) has been investigated, using a nisin-biogel combining the antimicrobial peptide (AMP) nisin with the natural polysaccharide guar-gum. Since in in vivo conditions bacteria may be exposed to decreased antimicrobial concentrations, known as subinhibitory concentrations (sub-MICs), effects of nisin-biogel sub-MIC values corresponding to 1/2, 1/4 and 1/8 of nisin’s minimum inhibitory concentration (MIC) on virulence expression by six *Staphylococcus aureus* DFI isolates was evaluated by determining bacteria growth rate; expression of genes encoding for staphylococcal protein A (*spA*), coagulase (*coa*), clumping factor A (*clfA*), autolysin (*atl*), intracellular adhesin A (*icaA*), intracellular adhesin D (*icaD*), and the accessory gene regulator I (*agrI*); biofilm formation; Coa production; and SpA release. Nisin-biogel sub-MICs decreased bacterial growth in a strain- and dose-dependent manner, decreased *agrI*, *atl* and *clfA* expression, and increased *spA*, *coa*, *icaA* and *icaD* expression. Biofilm formation increased in the presence of nisin-biogel at 1/4 and 1/8 MIC, whereas 1/2 MIC had no effect. Finally, nisin-biogel at sub-MICs did not affect coagulase production, but decreased SpA production in a dose-dependent manner. Results highlight the importance of optimizing nisin-biogel doses before proceeding to in vivo trials, to reduce the risk of virulence factor’s up-regulation due to the presence of inappropriate antimicrobial concentrations.

## 1. Introduction

Diabetes *mellitus* (DM) is a lifelong metabolic disorder that affects approximately 537 million people worldwide [1]. The development of diabetic foot ulcers (DFUs) is a serious complication associated with the DM triad of neuropathy, vasculopathy and immunopathy [2]. The severe loss of skin protective barriers creates a chance for tissue colonization by opportunistic microorganisms, including *Staphylococcus aureus*. Diabetic foot infections (DFIs) usually become chronic and result in increased patient morbidity and mortality, the most common DM complication requiring hospitalization and often resulting in lower-extremity amputations [3]. 

*S. aureus* is a Gram-positive bacterium that expresses several regulatory and virulence factors, which contributes to its success as a human opportunistic pathogen [4]. In the first step of a staphylococcal infection, the adhesion phase, the production of several cell surface-associated factors occurs, including clumping factor A (ClfA), staphylococcal protein A (SpA), and coagulase (Coa) that facilitate tissue attachment and evasion of the host immune system [4,5]. Staphylococcal major autolysin (Atl) is also relevant for staphylococcal attachment to surfaces, also participating in bacterial cell wall degradation, lysis mediated biofilm development and secretion of cytoplasmic proteins [6,7,8]. 

The ability to form biofilms is considered a major staphylococcal pathogenic feature [9]. Biofilms are characterized by the growth of adherent bacterial populations inside a self-produced matrix of extracellular polymeric substances, conferring this sessile mode of life survival advantages, including enhanced antimicrobial resistance. The intercellular adhesion (*ica*) locus, *icaADBC*, is associated with cell-to-cell adhesion, being responsible for the biosynthesis of the biofilm exopolysaccharide intercellular adhesion (PIA) [10,11,12,13]. The coordination between *S. aureus* adhesion and detachment is regulated by Quorum Sensing (QS). The accessory gene regulator (*agr*) of *S. aureus* is a QS global transcriptional regulator, controlling virulence factors and biofilm expression in a time and population density-dependent manner [10,14,15].

Another key factor for the success of *S. aureus* as a human pathogen is the ability to rapidly develop or acquire multiple antibiotic resistance determinants. Nowadays, the development and spread of pathogenic bacteria that are resistant to conventional antibiotics is a major public health concern, which makes it urgent to discover, develop, and implement new effective therapeutic strategies [16,17,18,19,20]. Antimicrobial peptides (AMPs) have been proposed as promising therapeutic candidates to inhibit bacterial growth which can be used synergistically with antibiotics [17]. One of the most studied AMPs is nisin, a lantibiotic produced by *Lactococcus lactis* which interacts with the bacterial cell wall precursor lipid II, inhibiting cell wall synthesis, and uses lipid II as a docking molecule for subsequent pore formation [21]. Nisin was first commercialized as a food preservative, being recognized as safe by the Food and Agriculture Organization/World Health Organization (FAO/WHO) [17,22,23]. In recent years, it started to be investigated in veterinary and pharmaceutical fields, including for the management of bacterial infections, as this polypeptide presents antimicrobial activity against a broad spectrum of Gram-positive bacteria [17]. *Staphylococcus* species have a remarkable susceptibility to nisin, which represent an advantage for nisin application towards skin infections, including DFIs, and the treatment of multiple drug resistant systemic infections [17,22]. 

As AMPs can be inactivated or degraded before achieving their target, different methods for AMPs delivery have been widely investigated, aiming at increasing their clinically efficacy. Natural polysaccharides have been considered promising drug delivery systems mainly due to their non-toxicity, sustainability, biodegradability, biocompatibility, abundance, availability, and cost-effectiveness [24]. Guar gum is a natural, uncharged, and water-soluble polysaccharide that has been largely used for targeted drug delivery, promoting a controlled drug release and availability [25,26]. Considering all the promising features of nisin and guar gum, a guar gum gel-based delivery system for nisin, nisin-biogel, has been developed by our research team as an alternative or complementary strategy to conventional antibiotics used in DFIs treatment [27]. Previous studies have shown that the nisin-biogel had inhibitory capacity towards *S. aureus* DFI isolates either in their planktonic and biofilm forms [27], could be applied in combination with conventional antibiotics and antiseptics to improve their efficacy [28,29], maintained its activity when stored at temperatures below 22 °C for 24 months [30], exhibited no significant levels of cytotoxicity on human keratinocyte cells [30] and was able to diffuse and keep its antimicrobial activity in a DFU collagen three-dimensional (3D) model established to mimic the DFU environment [29]. However, before proceeding to in vivo trials, the effect of drug therapeutic doses on virulence determinants expression by *S. aureus* must be addressed, as it may affect infection pathogenesis. Minimum inhibitory concentration (MIC) is defined as the lowest concentration of an antimicrobial that inhibits the growth of most of the target bacterial population, under controlled in vitro conditions [31]. However, in DFIs patients there is a poor diffusion of the antimicrobials that results from DM patient’s compromised blood circulation and low perfusion due to angiopathy [31,32]. As such, in in vivo infections, bacteria may be exposed to a reduced effective concentration of antimicrobials, referred to as subinhibitory concentrations (sub-MICs), which can lead to a wide variety of physiological and morphological effects on bacteria and, consequently, affect infection pathogenesis [31,33]. Besides promoting bacterial resistance, sub-MICs of antimicrobials can modulate the virulence of *S. aureus*, by influencing gene expression, biofilm production and the QS system, which may impact the outcome of staphylococcal infections [31,33,34,35,36]. As such, the present study evaluated the effects of nisin-biogel sub-MICs on *S. aureus* DFI isolates growth rate, virulence-related genes expression, namely of *agrI*, *spA*, *clfA*, *coa*, *atl*, *icaA* and *icaD*, ability to form biofilm, Coa production and SpA release, aiming at confirming its suitability for in vivo administration. 

## 2. Results

### 2.1. Effect of Nisin-Biogel Sub-MICs on S. aureus DFI Isolates Growth Rate 

**Figure 1:**
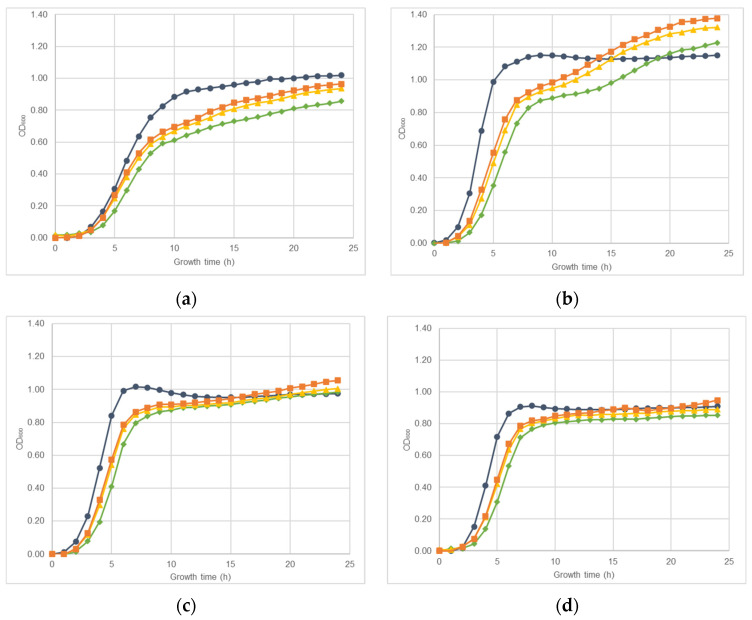

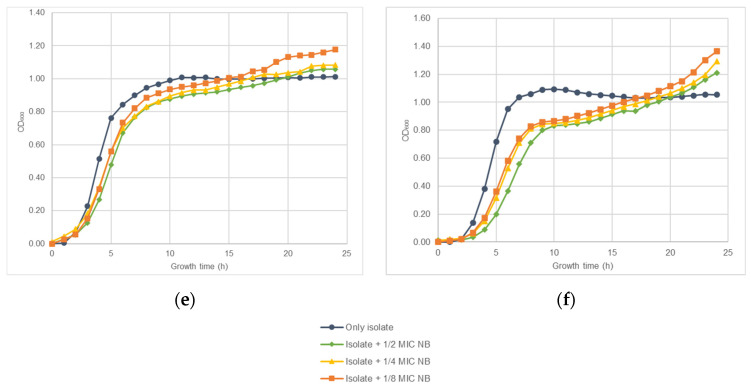
Growth curves obtained for *S. aureus* clinical isolates during 24 h at 37 °C with shaking (150 rpm) in the absence or in the presence of different sub-MICs of nisin-biogel. Results are presented as mean values of three independent assays, reflecting bacterial growth for each *S. aureus* clinical isolate under the different conditions tested. (**a**): Strain ID A 5.2, isolated from a DFI aspirate (*agrI* +; *spA* +; *atl* +; *clfA* +; *coa* +; *icaA* +; *icaD* +; Biofilm production +; MSSA). (**b**): Strain ID A 6.3, isolated from a DFI aspirate (*agrI* +; *spA* +; *atl* +; *clfA* +; *coa* +; *icaA* +; *icaD* +; Biofilm production +; MSSA). (**c**): Strain ID B 1.1, isolated from a DFI biopsy (*agrI* +; *spA* +; *atl* +; *clfA* +; *coa* +; *icaA* +; *icaD* +; Biofilm production +; MRSA). (**d**): Strain ID B 14.2, isolated from a DFI biopsy (*agrI* +; *spA* +; *atl* +; *clfA* +; *coa* +; *icaA* +; *icaD* +; Biofilm production +; MRSA). (**e**): Strain ID Z 1.1, isolated from a DFI swab (*agrI* +; *spA* +; *atl* +; *clfA* +; *coa* +; *icaA* +; *icaD* +; Biofilm production +; MRSA). (**f**): Strain ID Z 5.2, isolated from a DFI swab (*agrI* +; *spA* +; *atl* +; *clfA* +; *coa* +; *icaA* +; *icaD* +; Biofilm production +; MSSA). MIC: minimum inhibitory concentration. NB: nisin-biogel. OD_600_: optical density at 600 nm.

The effect of nisin-biogel sub-MICs on *S. aureus* DFI isolates A 5.2, A 6.3, B 1.1, B 14.2, Z 1.1, and Z 5.2 growth rate is represented in Figure 1. Both in the presence or absence of nisin-biogel at sub-MICs, all *S. aureus* clinical isolates growth curves presented the typical sigmoidal pattern with the three phases of bacterial growth curves—the lag phase, the exponential phase, and the stationary phase. Nevertheless, different clinical isolates showed different growth rates, with isolate A 6.3 having the highest growth rate, and isolate A 5.2 having the lowest one. Nisin-biogel at sub-MICs slowed bacterial growth, delaying the beginning of the exponential growth phase. Nisin-biogel concentration equivalent to 1/8 MIC was the one that promoted the lower change in bacterial growth, while the 1/2 MIC was the concentration that promoted a higher change on the bacterial growth. For some *S. aureus* clinical isolates, including isolates Z 5.2, A 6.3, B 1.1 and Z 1.1, there was an increase in bacterial growth in the stationary phase in the presence of nisin-biogel at sub-MICs. 

### 2.2. S. aureus DFI Isolates Gene Expression Kinetics 

**Figure 2:**
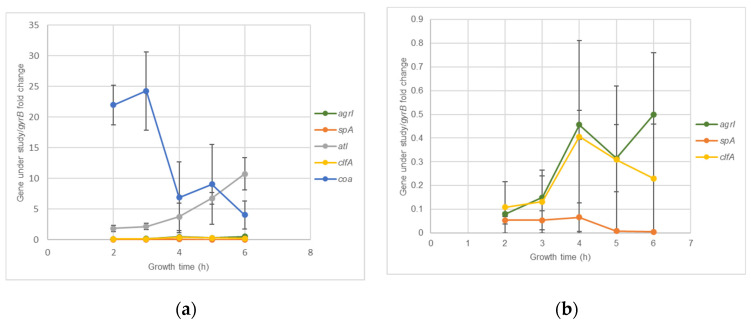
*agrI*, *spA*, *clfA*, *atl* and *coa* expression kinetics by *S. aureus* during a five hour growth period. Results are expressed as ‘gene under study/*gyrB*’ fold changes at 2, 3, 4, 5 and 6 h. Values are presented as means values ± SD (isolates A 5.2 and Z 5.2). (**b**) corresponds to an amplification of part of (**a**), so the y axis has different scales in the two figures. *agrI*: accessory gene regulator I; *spA*: gene encoding staphylococcal protein A; *atl*: gene encoding autolysin; *clfA*: gene encoding clumping factor A; *coa*: gene encoding coagulase; *gyrB*: gene encoding gyrase B.

**Figure 3:**
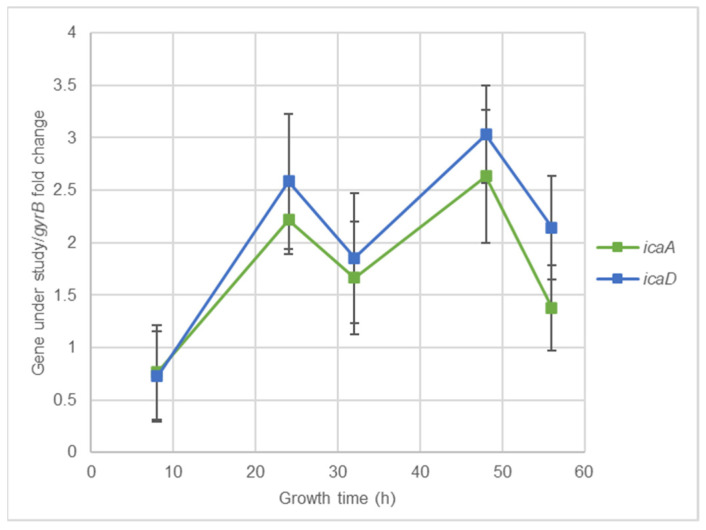
*icaA* and *icaD* expression kinetics for 48 h. Results are expressed as ‘gene under study/*gyrB*’ fold changes at 8, 24, 32, 48, and 56 h. Values are presented as means values ± SD (isolates A 5.2 and Z 5.2). *icaA*: gene encoding intracellular adhesin A; *icaD*: gene encoding intracellular adhesin D; *gyrB*: gene encoding gyrase B.

*S. aureus* clinical isolates virulence genes expression kinetics was accessed using RT-qPCR assays. Figure 2a,b show that for *agrI* and *atl*, the highest expression levels were reached at 6 hours’ incubation, while for *spA* and *clfA* it was at 4 h. For *coa*, maximum expression levels were reached at 3 h. Although the optical expression of the different genes occurred at different periods of bacterial growth, all revealed a considerable expression at 4 h’ incubation, which allowed to select this time point as the more adequate for further evaluation of the effects of nisin-biogel sub-MICs on *agrI*, *spA*, *clfA*, *atl* and *coa* expression. Genes *icaA* and *icaD* reach their maximum expression levels at 48 h (Figure 3), which allowed to select this time point to *icaA* and *icaD* expression assays. 

### 2.3. Effect of Nisin-Biogel at Sub-MICs on Gene Expression by S. aureus DFI Isolates

**Figure 4:**
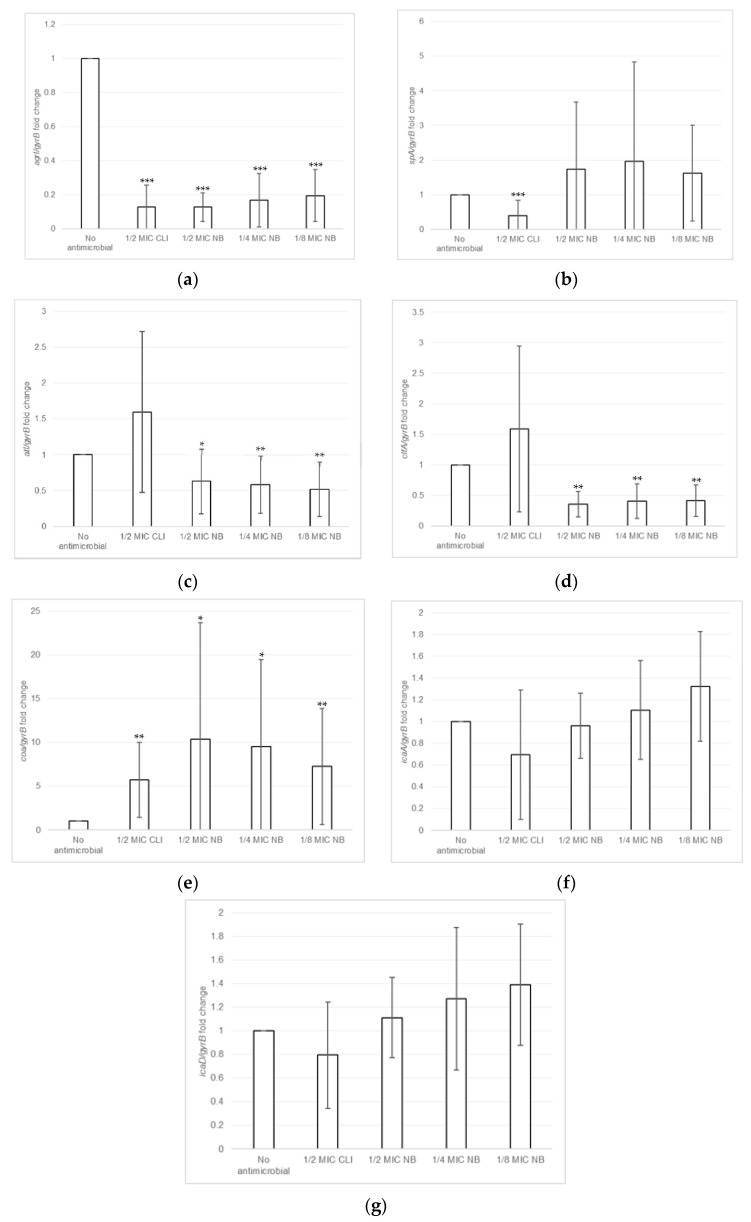
(**a**–**g**): Effects of nisin-biogel at 1/2 MIC, 1/4 MIC and 1/8 MIC and clindamycin at 1/2 MIC on *agrI*, *spA*, *atl*, *clfA*, *coa*, *icaA* and *icaD* mRNA expression, respectively. Results are expressed as n-fold differences in the ‘gene under study/*gyrB*’ ratio in the presence of the different conditions described above relative to ‘gene under study/*gyrB*’ ration in the growth control (no antimicrobial). Values are means ± SD (two repeated different experiments). Asterisks indicate statistically significant differences between treatments and control (* = *p* < 0.05; ** = *p* < 0.01; *** = *p* < 0.001). NB: nisin-biogel; CLI: clindamycin; MIC: minimum inhibitory concentration. *agrI*: accessory gene regulator I; *spA*: gene encoding staphylococcal protein A; *atl*: gene encoding autolysin; *clfA*: gene encoding clumping factor A; *coa*: gene encoding coagulase; *icaA*: gene encoding intracellular adhesin A; *icaD*: gene encoding intracellular adhesin D; *gyrB*: gene encoding gyrase B.

The effect of nisin-biogel and clindamycin at sub-MICs on virulence genes expression by *S. aureus* DFI isolates was investigated using RT-qPCR assays. As shown in Figure 4 and Table S1 (available as Supplementary Data), the effects depended on the virulence factor, on the *S. aureus* clinical isolate, and on the subinhibitory concentration under study. Overall, nisin-biogel and clindamycin at sub-MICs values significantly decreased the expression of *agrI*, with nisin-biogel at 1/2 MIC being the one that reduced the expression of *agrI* the most, and 1/8 MIC the one that least reduce *agrI* expression (Figure 4a). Nisin-biogel at sub-MICs increased the expression of *spA*, while clindamycin at 1/2 MIC significantly decreased the expression of this gene (Figure 4b). For *atl*, nisin-biogel at sub-MICs significantly decreased its expression, with 1/2 MIC being the one that least decreased *atl* expression. Unlike nisin-biogel at sub-MICs, clindamycin at 1/2 MIC increased the expression of this gene (Figure 4c). Nisin-biogel at sub-MICs significantly decreased *clfA* expression, with 1/2 MIC being responsible for the highest increase in the expression of this gene. On the other hand, clindamycin at 1/2 MIC significantly increased *clfA* expression (Figure 4d). For *coa*, both nisin-biogel and clindamycin at sub-MICs increased gene expression and, regarding nisin-biogel, 1/2 MIC was the one that increased *coa* expression the most (Figure 4e). For *icaA*, nisin-biogel at 1/4 and 1/8 MIC slightly increased the expression of this gene, while at 1/2 MIC slightly decreased *icaA* expression. Clindamycin at 1/2 MIC decreased *icaA* expression (Figure 4f). Finally, for *icaD*, nisin-biogel sub-MICs slightly increased gene expression, while clindamycin at 1/2 MIC decreased its expression (Figure 4g).

### 2.4. Effect of Nisin-Biogel Sub-MICs on the Ability of S. aureus DFI Isolates to Form Biofilm

**Figure 5:**
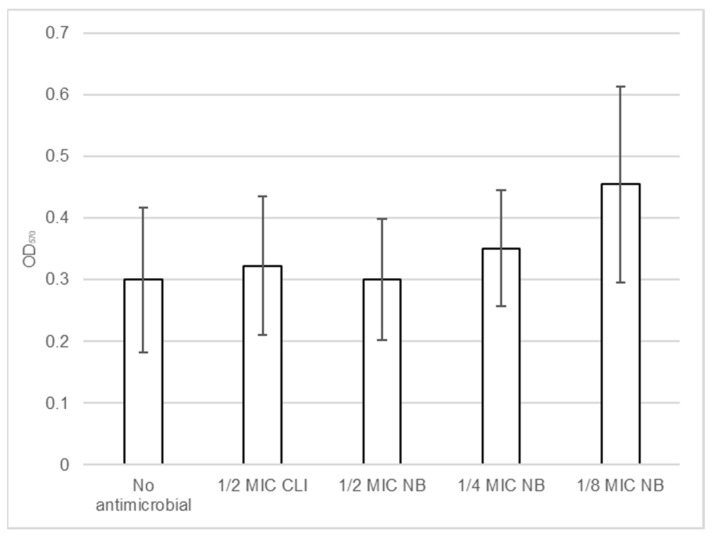
Effects of nisin-biogel at 1/2 MIC, 1/4 MIC and 1/8 MIC and clindamycin at 1/2 MIC on the ability of *S. aureus* DFI isolates to form biofilm. Values are presented as means ± SD (three repeated different experiments). Asterisks indicate statistically significant differences between treatments and control. NB: nisin-biogel; CLI: clindamycin; MIC: minimum inhibitory concentration. OD_570_: optical density at 570 nm.

Biofilm formation by *S. aureus* DFI isolates in the presence of nisin-biogel and clindamycin at sub-MICs was determined using a microtiter assay. Results shown in Table S2 (available as Supplementary Data) demonstrate that different *S. aureus* clinical isolates have different responses to nisin-biogel and clindamycin at sub-MICs. In Figure 5 it is possible to observe that, overall, nisin-biogel at 1/4 and clindamycin at 1/2 MIC exhibit a trend to increase the ability of *S. aureus* isolates to form biofilm, and, in the presence of nisin-biogel at 1/8 MIC, biofilm formation significantly increases. Oppositely, nisin-biogel at 1/2 MIC had no effect in the ability of *S. aureus* clinical isolates to form biofilm. 

### 2.5. Effect of Nisin-Biogel Sub-MICs on Coa Production by S. aureus DFI Isolates

Coagulase test was used to monitor coagulase production by *S. aureus* clinical isolates in presence and absence of nisin-biogel and clindamycin at sub-MICs. Results were considered valid if the control plasma showed no signs of clotting in all assays. 

Different clinical isolates showed different coagulase production ability over 4 h and after 24 h of incubation in the presence or absence of nisin-biogel and clindamycin at sub-MICs, as shown in Tables S3 and S4 (available as Supplementary Data). Besides depending on the *S. aureus* clinical isolate, the effects of antimicrobials sub-MICs on coagulation varied according to the bacterial growth period. During the first 4 h, in which results were monitored on an hourly basis, clotting in the absence of nisin-biogel was higher than or equal to the one obtained in the presence of nisin-biogel at sub-MICs. After 24 h incubation, most clinical isolates showed the same degree of clotting in the presence or absence of nisin-biogel. Regarding the effect of clindamycin at 1/2 MIC, for isolates A 5.2, A 6.3, and Z 5.2 there was no signs of coagulation in the presence of the antibiotic, whereas for isolates B 1.1, B 14.2 and Z 1.1, clotting degree was equal or higher than the one obtained in the absence of clindamycin at 1/2 MIC. 

### 2.6. Effect of Nisin-Biogel Sub-MICs on SpA Release by S. aureus DFI Isolates 

**Figure 6:**
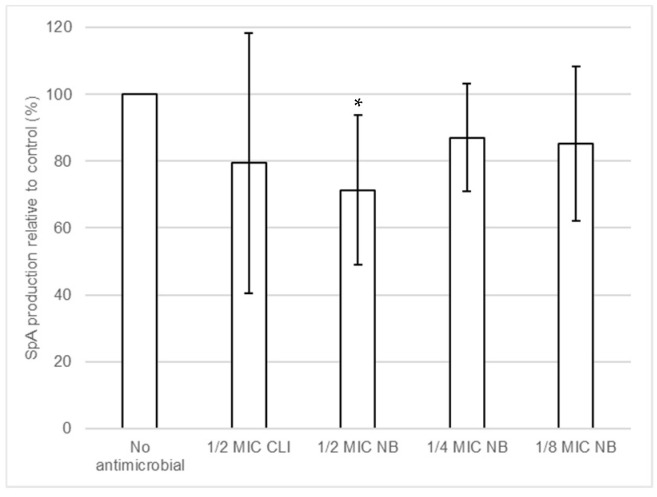
Effects of nisin-biogel at 1/2 MIC, 1/4 MIC and 1/8 MIC and clindamycin at 1/2 MIC on SpA production by *S. aureus* DFI isolates. Results are the ratios of the amount of SpA (pg/mL) in the bacterial supernatants incubated with nisin-biogel and clindamycin to the mean amount of SpA (pg/mL) incubated without antimicrobials and are expressed as supernatants. Values are presented as mean values ± SD. Asterisks indicate statistically significant differences between treatments and control (* = *p* < 0.05). NB: nisin-biogel; CLI: clindamycin; MIC: minimum inhibitory concentration. SpA: staphylococcal protein A.

The effect of nisin-biogel and clindamycin at sub-MICs on SpA release by *S. aureus* DFI isolates was investigated using Protein A ELISA Kit. Results shown in Table S5 (available as Supplementary Data) demonstrate that different *S. aureus* clinical isolates have different responses regarding SpA release in the presence of antimicrobials sub-MICs. Figure 6 shows the overall effects of nisin-biogel and clindamycin at sub-MICs on the release of SpA by *S. aureus* DFI isolates. Nisin-biogel at 1/2 significantly decreased the release of SpA, and the other conditions under study exhibited a trend to decrease SpA release by *S. aureus* clinical isolates. 

## 3. Discussion

Diabetes *mellitus* (DM) is one of the most disseminated chronic diseases worldwide. The last International Diabetes Federation (IDF) Atlas states that, in 2021, 537 million adults aged over 20 years were living with diabetes worldwide. In Portugal, there were over 990 million cases of diabetes registered this year, with the cost associated to each diabetic patient being estimated to be approximately 2.990 euros (https://diabetesatlas.org/data/en/country/159/pt.html, accessed on 27 November 2021), proving that diabetes represents a significant health burden in our country [37].

A frequent and most devastating consequence of diabetes is the development of diabetic foot ulcers (DFU), which in about 50% of the cases become infected, developing to diabetic foot infections (DFI) [38]. Besides the conventional therapeutic protocols available for DFI treatment (e.g., debridement, wound healing agents, surgery and antibiotic therapy), several advanced therapeutics are also available, including bacteriophage therapy, negative-pressure wound therapy, hyperbaric oxygen therapy, stem cell therapy and off-loading [38]. Antimicrobial peptides are also a promising approach for DFI treatment; however, as observed for conventional antibiotics, angiopathy and low perfusion associated with DFU pathogenesis may prevent antimicrobials to reach the infected DFUs at effective concentration, leading to the presence of sub-MICs at the site of infection, which may impair treatment success [31,32].

The presence of antimicrobials sub-MICs can have several effects on bacteria, including the inhibition of bacterial growth [39,40] and therefore on infection progression [35,39]. As such, the effects of nisin-biogel at sub-MICs on bacterial growth were assessed. Previously, Field et al. [17] showed that nisin at sublethal concentrations slightly increased the lag period of *S. aureus* growth curve, which supports the results from this study, as the nisin-biogel sub-MICs values tested caused a reduction on *S. aureus* DFI isolates growth by increasing the lag phase. This reduction occurred in a dose-dependent manner, with the nisin-biogel at 1/2 MIC being the concentration that decreased bacterial growth the most, whereas 1/8 MIC was the one that least affected bacterial growth. In the stationary growth phase, the *S. aureus* clinical isolates Z 5.2, A 6.3, B 1.1 and Z 1.1 seem capable of adapting to the presence of nisin-biogel at sub-MICs, presenting an increase in growth rate. 

*S. aureus* expresses a multitude of virulence factors in a coordinated manner, and many of them are under the control of the *agr* quorum sensing system. The *agr* gene positively controls the expression of many exotoxins, mainly produced after the end of the exponential growth phase, allowing bacteria to spread from the colonization sites to deeper tissues, and negatively controls the transcription of some cell wall-associated proteins, mainly synthesized during exponential growth [41]. According to our results, *agrI* expression levels by the *S. aureus* isolates tested only started to be relevant after 4 h, a period during which bacterial adherence to the host tissues already occurred in vivo. At this time point, the cell-surface proteins start to be down-regulated, whereas the virulence genes associated with bacterial dissemination and biofilm formation start to be up-regulated [4,42,43]. Peng et al. [44] showed that *agr* activity is required for post-exponential phase expression of various secreted proteins, which supports the fact that in the present study *agr* mRNA expression has its peak at 6 h.

SpA is a microbial surface protein that plays an important role in interfering with host defenses, inhibiting antibody-mediated phagocytosis. Staphylococcal extracellular protein, Coa, also contributes for the persistence of *S. aureus* in the host cell, by stimulating clotting formation in plasma, inhibiting host clearance mechanisms [45,46]. Both proteins are mainly expressed in the early stage of *S. aureus* growth, i.e., before 3–4 h. A study by Vandenesch et al. [47] suggested that *spA* mRNA is synthetized for a brief period in the beginning of the exponential phase and then is switched off. Moreover, according to Lebeau et al. [48], *coa* mRNA is mainly expressed at the early stages of *S. aureus* growth. Results from this study also showied that *coa* and *spA* mRNA are mostly expressed at the early exponential growth phase. 

ClfA is also a staphylococcal surface protein that binds to Fg, allowing bacteria to colonize traumatized tissue and, later, form biofilms [49]. *clfA* is mainly expressed after 4 h of bacterial growth, i.e., at the late exponential growth phase. Results by Josefsson et al. [50] show that *clfA* expression is higher at 6 h and remains high until 24 h of *S. aureus* growth, which is not entirely in line with what we obtained in the present study, suggesting that different *S. aureus* strains may have different *clfA* mRNA expression kinetics. Atl protein has a multitude of functions, including attachment, bacterial cell wall degradation, biofilm development and bacteriolytic activity [51]. In the present study, *atl* expression increased with *S. aureus* growth period, within a time frame of 6 h, probably due to the increased growth rate over this time period. These results are in accordance with those by Oshida et al. [52] that also reported an increase in *atl* expression during the exponential growth phase.

One of the most relevant staphylococcal virulence factors is biofilm production, conferring bacteria a wide range of adaptive advantages that contribute to bacteria survival and persistence in the host [32]. Thus, *icaA* and *icaD*, involved in *icaADBC*-dependent biofilm formation, are mainly expressed latter during *S. aureus* growth. According to Atshan et al., 2013, *icaA* and *icaD* are up-regulated at 24 h of *S. aureus* growth and not at 48 h as observed in the present study, which suggests that *ica* mRNA expression kinetics may differ between *S. aureus* strains [53]. Furthermore, Patel et al. [54] evaluated gene expression during *Staphylococcus epidermidis* biofilm formation and, in accordance with the present study, the expression levels of *icaA* and *icaD* at 48 h were the highest ones. Although this study focused on *S. aureus*, *S. epidermidis* also produces biofilm in an *icaADBC*-dependent manner, allowing to suggest that the time frame required for the expression of *icaA* and *icaD* may be identical in both species.

Several studies have shown that the use of antimicrobials at sub-MICs modulates virulence gene expression and, consequently, influences bacterial pathogenicity [21,39]. Determining the effects of antimicrobials sub-MICs on virulence genes expression may provide important information for the rational use of antimicrobials in clinical practice [39]. One of the antibiotics currently applied as a DFIs alternative treatment is clindamycin, used in this study as a control to compare the effects of nisin-biogel at sub-MICs on *S. aureus* gene expression [21,55].

Subinhibitory levels of nisin-biogel decreased the expression of the regulatory gene *agrI* in a dose-dependent manner, which could lead to changes in the virulence modulation and positively affect infection treatment [10,14,15]. Regarding *spA* and *coa*, nisin-biogel at sub-MICs exhibited a trend to increase the expression of these genes, which can negatively affect infection treatment, as both SpA and Coa contribute for bacterial evasion from the host immune system [4,5]. Nisin-biogel at sub-MICs significantly decrease the expression of *atl* and *clfA*, possibly reducing *S. aureus* pathogenic potential. These results are in accordance with those by Zhao et al. [56], which stated that the exposure of *S. aureus* for 1 h to a nisin concentration equivalent to 1/2 MIC lead to a down-regulation of *atl* and *clfA*.

Concerning *icaA* and *icaD*, involved in biofilm formation, the nisin-biogel at subinhibitory concentrations slightly increased their expression, which can contribute for an increase in biofilm formation, hindering infection treatment. Moreover, the lower the concentration of nisin-biogel, a higher increase of *icaA* and *icaD* mRNA levels was observed, which reinforces the importance of defining the optimum dosage of antimicrobials to be applied to the treatment of bacterial infections.

The effects of subinhibitory concentrations on bacterial biofilm formation were also investigated. Biofilms play a major role in the pathogenesis of *S. aureus*, and are present in 60 to 100% of chronic wounds, including DFUs [57]. Several studies have shown that sub-MICs of some antimicrobials can affect bacterial biofilm formation in vitro, which may have clinical importance [58,59]. Angelopoulou et al. [60] observed that nisin at 1/2, 1/4 and 1/8 MIC significantly increased biofilm formation by *S. aureus*, with the concentration of 1/8 MIC also being the one that most influenced biofilm formation, as observed in this study. When exposed to nisin-biogel at 1/4 and 1/8 MIC values, the ability of *S. aureus* clinical isolates to form biofilm increased in a dose-dependent manner, while 1/2 MIC had no effect on biofilm formation. Moreover, the lower the concentration of nisin-biogel, the higher the increase in biofilm formation, which is mostly consistent with the effect on the expression of *icaA* and *icaD*. Moreover, as stated by other authors, the increase of biofilm formation may be due to the fact that sublethal concentrations of antimicrobials are cell stressors, which can enhance the production of biofilm matrix polymers [59,61]. However, Andre et al. [58] reported that subinhibitory concentrations of nisin promoted a reduction in biofilm formation, which suggests that different strains may present different responses regarding biofilm formation in the presence of sub-MICs of this antimicrobial peptide.

Alterations in the virulence determinants mRNA levels in the presence of subinhibitory levels of antimicrobials do not always result in changes in protein synthesis or functional activity [62,63]. According to our results, the increase in quantification of *spA* and *coa* mRNA in the presence of nisin-biogel at sub-MICs are not directly associated with an increase in SpA production or Coa activity, respectively. In fact, there was an unexpected significant increase of *coa* expression in the presence of subinhibitory levels of nisin-biogel. When accessing coagulase production, results were different, as most of the clinical isolates showed a similar coagulase activity in the presence of nisin-biogel at sub-MICs. Thus, our findings suggest that *coa* expression increase that occurs in the presence of nisin-biogel at sub-MICs may be associated with an increase in mRNA stabilization, as previously suggested by Blickwede et al. [64] regarding clindamycin influence on *coa* stability. On another end, SpA protein levels exhibited a trend to decrease in the presence of nisin-biogel at sub-MICs, which is partly not consistent with what happened to SpA mRNA levels. Actually, in the presence of nisin-biogel at sub-MICs levels, *S. aureus* DFI isolates increased SpA mRNA levels, which seems to suggest that the inhibition of virulence expression by nisin-biogel is primarily due to the blockage of protein translation at the ribosome, rather than the inhibition of virulence genes transcription.

## 4. Materials and Methods

### 4.1. Bacterial Strains

*S. aureus* isolates used in the present study were previously obtained from patients with clinically infected DFUs using biopsies (isolates with a B in their identification code), swabs (isolates with a Z in their identification code) or aspirates (isolates with an A in their identification code), according to the current clinical guidelines [65]. The isolates were virulence and antimicrobial resistance profiles were previsouly analyzed by Polymerase Chain Reaction (PCR), including their biofilm-forming ability, and their clonal profile was previously determined by Pulse Field Gel Electrophoresis (PFGE) and Multilocus Sequence Type (MLST) [66]. *S. aureus* isolates A 5.2, A 6.3, B 1.1, B 14.2, Z 1.1 and Z 5.2 were selected for this study due to their virulence traits, which includes the presence of genes encoding for staphylococcal protein A (*spA*), autolysin (*atl*), clumping factor A (*clfA*), coagulase (*coa*), intracellular adhesin A (*icaA*), intracellular adhesin D (*icaD*) and the accessory regulator gene I (*agrI*), and to their capacity to produce biofilms. Moreover, isolates B 14.2, B 1.1 and Z 1.1 are methicillin-resistant *S. aureus* (MRSA), whereas isolates A 5.2, A 6.3 and Z 5.2 are methicillin-susceptible *S. aureus* (MSSA) [38,66]. The MICs of nisin-biogel and clindamycin for the *S. aureus* clinical isolates were previously determined, being, in average, of 22.5 µg/mL and 0.033 µg/mL, respectively [27,67].

### 4.2. Antimicrobial Solutions

A stock solution of nisin (1000 µg/mL) was obtained by dissolving 1 g of nisin powder (2.5% purity, 1000 IU/mg, Sigma-Aldrich, St. Louis, MO, USA) in 25 mL of HCl (0.02 M) (Merck, Darmstadt, Germany). This solution was sterilized by filtration through a 0.22 µm cellulose acetate membrane filter (Millipore, Burlington, MA, USA) and stored at 4 °C [27].

Brain Heart Infusion (BHI) or Trypticase Soy Broth (TSB) with guar-gum gel at 0.75% (*w*/*v*) were prepared by dissolving 3.75 g of guar-gum (Sigma-Aldrich, St. Louis, MO, USA) and 18.5 g of BHI powder (VWR Chemicals, Leuven, Belgium) or 15 g of TSB powder (VWR Chemicals, Leuven, Belgium), respectively, in 500 mL of sterile distilled water, and heat sterilized by autoclave [27].

Clindamycin is an alternative antibiotic currently used in clinical practice associated with mild DFIs and was used in the present study at 1/2 MIC as a control for comparing the effects of nisin-biogel at sub-MICs on *S. aureus* DFI isolates virulence factors expression [28]. A stock solution of clindamycin was obtained by dissolving 6.6 mg of clindamycin powder (Sigma-Aldrich, St. Louis, MO, USA) in 10 mL of sterile water and filtered using a 0.22 μm cellulose acetate membrane filter. This stock solution was kept frozen at −80 °C and diluted with sterile water to the final concentration of 0.0165 µg/mL when required.

### 4.3. Effects of Nisin-Biogel Sub-MICs on S. aureus DFI Isolates Growth Rate

*S. aureus* DFI isolates were inoculated in a non-selective BHI agar medium (VWR Chemicals, Leuven, Belgium) at 37 °C for 24 h. After incubation, bacterial suspensions of 10^8^ CFU/mL were prepared directly from plate cultures using a 0.5 McFarland standard in NaCl (Merck, Damstrants, Germany), and the bacterial suspensions were diluted in fresh BHI broth or in fresh BHI broth containing guar gum at 0.75% (*w*/*v*) to a final concentration of 10^7^ CFU/mL. Afterwards, nisin was added to the fresh BHI broth with guar gum to obtain bacterial cultures with nisin-biogel at 1/2, 1/4 and 1/8 MIC values. Then, the wells of a 96-well flat-bottomed polystyrene microtiter plates (Thermo Scientific, Waltham, MA, USA) were inoculated with 200 µL of the negative controls (fresh BHI broth and fresh BHI broth with guar gum at 7.5 mg/mL) and with 200 µL of the different bacterial suspensions previously prepared, namely in fresh BHI broth, BHI broth with guar gum plus 1/2 MIC of the nisin-biogel ((nisin) = 11.25 µg/mL; (guar gum) = 7.5 mg/mL), BHI broth with guar gum plus 1/4 MIC of the nisin-biogel ((nisin) = 5.625 µg/mL; (guar gum) = 7.5 mg/mL) and BHI broth with guar gum plus 1/8 MIC of the nisin-biogel ((nisin) = 2.8175 µg/mL; (guar gum) = 7.5 mg/mL).

Each different growth condition was evaluated in triplicate wells on three independent assays. During the 24 h of incubation at 37 °C with shaking (150 rpm), optical density at 600 nm (OD_600_) for each well were obtained automatically every hour, using the FLUOstar OPTIMA (BMG LABTECH, Ortenberg, Germany) microplate reader. For each isolate, OD_600_ was calculated by subtracting the average OD_600_ of the three blank wells (fresh BHI broth or fresh BHI broth with guar gum at 7.5 mg/mL) from the average of OD_600_ of the three replicates of the sample under evaluation. Final result corresponds to the average results of each independent assay replicates.

### 4.4. S. aureus DFI Isolates Gene Expression Kinetics

Isolates A 5.2 and Z 5.2 were inoculated in a non-selective BHI agar medium at 37 °C for 24 h. After incubation, bacterial suspensions of 10^8^ CFU/mL were prepared as described. Bacterial suspensions were diluted in fresh BHI broth to a final concentration of 10^7^ CFU/mL and grown at 37 °C with gyratory shaking (180 rpm). For quantification of *agrI*, *spA*, *coa*, *clfA* and *atl* expression, aliquots were collected after 2, 3, 4, 5 and 6 h of incubation for subsequent stabilization of total RNA with RNAprotect^®^ Bacteria Reagent (Qiagen, Hilden, Germany), enzymatic lysis of bacteria with Buffer TE containing lysostaphin (Sigma-Aldrich, St. Louis, MO, USA), RNA extraction using the Qiagen RNeasy Mini Kit (Qiagen, Hilden, Germany), and cDNA synthesis with random primers using a Promega Go ScriptTM Reverse Transcription System (Promega, Madison, WI, USA), according to manufacturer’s instructions. Gene expression was analyzed by RT-qPCR [68], using the specific primers shown in Table S6 (available as Supplementary Data). RT-qPCR was performed in a 7300 Real Time PCR System (Applied Biosystems, Waltham, MA, USA) using the following conditions: an initial uracil-N-glycosylase gene (UNG) activation at 50 °C for 2 min, followed by an initial DNA polymerase activation at 95 °C for 10 min, and 35 cycles consisting in melting at 95 °C for 15 s and annealing/extending at 60 °C for 1 min. A set of dissociation steps was also performed, using the following conditions: 95 °C for 15 s, 60 °C for 1 min, 95 °C for 15 s, and 60 °C for 15 s. For *icaA* and *icaD* genes, aliquots were collected after 8, 24, 32, 48 and 56 h of incubation for subsequent stabilization of total RNA, enzymatic lysis of bacteria, RNA extraction, cDNA synthesis and analyses by RT-qPCR. The relative standard curve method was used to quantify gene transcription, using gyrase B (*gyrB*) gene for normalization. An average ± standard deviation of the fold change obtained for the isolates A 5.2 and Z 5.2 was considered for the determination of gene expression kinetics and, consequently, the best growth time for further investigate the effects of nisin-biogel at sub-MICs on virulence-related genes expression.

### 4.5. Effects of Nisin-Biogel Sub-MICs on Gene Expression by S. aureus DFI Isolates

Isolates A 5.2, A 6.3, B 1.1, B 14.2, Z 1.1 and Z 5.2 were inoculated in a non-selective BHI agar medium at 37 °C for 24 h. After incubation, bacterial suspensions of 10^8^ CFU/mL were prepared as described. For each isolate, 5 different bacterial suspensions were prepared: in BHI broth, BHI broth plus clindamycin at 1/2 MIC ((clindamycin) = 0.0165 µg/mL), BHI broth with guar-gum at 7.5 mg/mL plus 1/2 MIC of the nisin-biogel ((nisin) = 11.25 µg/mL), BHI broth with guar-gum at 7.5 mg/mL plus 1/4 MIC of the nisin-biogel ((nisin) = 5.625 µg/mL) and in BHI broth with guar-gum at 7.5 mg/mL plus 1/8 MIC of the nisin-biogel ((nisin) = 2.8175 µg/mL). All these suspensions were incubated at 37 °C for 4 h to *agrI*, *spa*, *atl*, *coa* and *clfa* genes expression studies, and for 48 h to *icaA* and *icaD* genes expression studies, with gyratory shaking (180 rpm).

After incubation, stabilization of total RNA, enzymatic lysis of bacteria, RNA extraction and cDNA synthesis were performed, and the resulting cDNA was used as a template for RT-qPCR using the specific primers shown in Table S1 (available as Supplementary Data), and the relative standard curve method was used to quantify transcription. Therefore, to determine the effects of nisin-biogel and clindamycin sub-MICs on virulence-related genes expression, the expression levels of the genes under investigation were expressed as fold change of the *spa/gyrB*, *agrI/gyrB*, *coa/gyrB*, *clfa/gyrB*, *icaA/gyrB*, *icaD/gyrB* and *atl/gyrB* ratios in the presence of antimicrobials (nisin-biogel or clindamycin) relative to the *spa/gyrB*, *agrI/gyrB*, *coa/gyrB*, *clfa/gyrB*, *icaA/gyrB*, *icaD/gyrB* and *atl/gyrB* ratios, respectively, of the growth control (no antimicrobial present). For each isolate and each incubation condition, two different and independent assays were performed.

### 4.6. Effect of Nisin-Biogel Sub-MICs on the Ability of S. aureus DFI Isolates to Form Biofilm

To test the influence of nisin-biogel sub-MICs on biofilm formation, a modified version of the protocol described by Santos et al. [27] was performed. Isolates A 5.2, A 6.3, B 1.1, B 14.2, Z 1.1 and Z 5.2 were inoculated in a non-selective BHI agar medium at 37 °C for 24 h. Then, three to five colonies were collected using a sterile loop, resuspended in 5 mL of TSB and incubated for 18 h at 37 °C. After incubation, the turbidity of bacterial suspension was adjusted to 0.5 McFarland standard (10^8^ CFU/mL), and 1:100 dilutions were made in TSB with 0.25% glucose, TSB with 0.25% glucose plus 1/2 MIC of clindamycin ((clindamycin) = 0.0165 µg/mL), TSB with guar-gum at 7.5 mg/mL and 0.25% glucose plus 1/2 MIC of nisin-biogel ((nisin) = 11.25 µg/mL), TSB with guar-gum at 7.5 mg/mL and 0.25% glucose plus 1/4 MIC of nisin-biogel ((nisin) = 5.625 µg/mL) and TSB with guar-gum at 7.5 mg/mL and 0.25% glucose plus 1/8 MIC of nisin-biogel ((nisin) = 2.8175 µg/mL).

Bacterial suspensions were transferred to a sterile 96-well polystyrene plate (200 µL/well) and incubated at 37 °C for 48 h. After incubation, the content of each well was removed, and the wells were carefully washed three times with 180 µL of PBS, pH 7.0. Then, wells were filled with 200 µL of PBS, pH 7.0, and the microtiter plate was incubated in an ultrasound bath (Grant MXB14), at 50 Hz for 15 min, in order to disperse the biofilm-based bacteria from the microtiter plate surface. Finally, the OD of the suspension from each well was measured at 570 nm using the FLUOstar OPTIMA microplate reader. Results were calculated by subtracting the average OD_570_ of the three replicas of the negative controls (TSB broth or TSB broth with guar gum at 7.5 mg/mL) from the average of OD_570_ of the three wells of each sample. Final result corresponds to the average of the three independent assays.

### 4.7. Effect of Nisin-Biogel Sub-MICs on Coa Production by S. aureus DFI Isolates

Isolates A 5.2, A 6.3, B 1.1, B 14.2, Z 1.1 and Z 5.2 were inoculated in a non-selective BHI agar medium at 37 °C for 24 h. After, isolates were incubated in BHI broth, BHI broth plus clindamycin at 1/2 MIC ((clindamycin) = 0.0165 µg/mL), BHI broth with guar-gum at 7.5 mg/mL plus nisin-biogel at 1/2 MIC ((nisin) = 11.25 µg/mL), BHI broth with guar-gum at 7.5 mg/mL plus nisin-biogel at 1/4 MIC ((nisin) = 5.625 µg/mL) and BHI broth with guar-gum at 7.5 mg/mL plus nisin-biogel at 1/8 MIC ((nisin) = 2.8175 µg/mL) for 24 h or for 4 h at 37 °C. Coagulase test was performed by adding 0.1 mL of each culture to 0.3 mL of rabbit plasma previously rehydrated with sterile water. After gentle mixing, suspensions were incubated at 37 °C and examined every hour for 4 h, and after 24 h. Results were interpreted according to the scale proposed by Sperber & Tatini, 1975, where negative means no evidence of fibrin formation, positive 1+ means small unorganized clots, positive 2+ means small organized clot, positive 3+ means large organized clot, and positive 4+ means that the entire content of tube coagulates and is not displaced when tube is inverted [69]. As negative controls, 0.1 mL of BHI broth or 0.1 mL of BHI broth with guar-gum at 7.5 mg/mL were added to 0.3 mL of rabbit plasma and incubated without bacteria in the same conditions.

### 4.8. Effect of Nisin-Biogel Sub-MICs on SpA Release by S. aureus DFI Isolates

Isolates A 5.2, A 6.3, B 1.1, B 14.2, Z 1.1 and Z 5.2 were inoculated in a non-selective BHI agar medium at 37 °C for 24 h. After, isolates were incubated in BHI broth for 4 h, i.e., isolates were grown to the exponential phase at 37 °C. Then, the cultures were incubated in the presence of nisin-biogel at 1/2 MIC ((nisin) = 11.25 µg/mL), 1/4 MIC ((nisin) = 5.625 µg/mL) and 1/8 MIC ((nisin) = 2.8175 µg/mL), and clindamycin at 1/2 MIC ((clindamycin) = 0.0165 µg/mL) for 18 h at 37 °C with shaking. After incubation, bacterial suspensions were centrifuged at 1500× *g* rpm for 10 min at 4 °C, and supernatant were used to determine SpA level using the SpA ELISA Kit (Abcam, Cambridge, UK), as recommended by the manufacturer. Samples and standards were added to the 96-well plate, the assay was performed and absorvance values at 450 nm determined, as these values are directly proportional to the level of protein A in the sample. The results correspond to the ratios of the amount of SpA (pg/mL) in the bacterial supernatants incubated with clindamycin or nisin-biogel and the mean amount of SpA (pg/mL) in the bacterial supernatants incubated without antimicrobials and are expressed as percentages.

### 4.9. Statistical Analysis

Statistical analysis was carried out using Microsoft Excel 2016^®^. Quantitative variables are expressed as mean values ± standard deviation. Comparisons between treatments and control were performed using two-tailed Student’s *t*-tests. A confidence interval of 95% was considered, and *p*-values < 0.05 indicate statistical significance.

## 5. Conclusions

*S. aureus* produces a wide variety of virulence factors, such as adherence and colonization molecules, exotoxins, and enzymes, and forms biofilms, which contribute to its ability to colonize host tissues and cause disease, making it difficult to control staphylococcal infections. The effect of antimicrobial agents on these virulence factors’ expression has become a major focal point in the study of new antimicrobial alternatives. The present results demonstrated that nisin-biogel at subinhibitory levels affects the growth of *S. aureus* in a strain-dependent and dose-dependent manner, as well as the production of several virulence factors, including coagulase, protein A, and biofilm. The expression of some virulence-related genes, such as *agrI*, *atl* and *clfA*, were found to be repressed by nisin-biogel at sub-MICs, whereas the transcription levels of *spA*, *coa*, *icaA* and *icaD* were increased. Results highlight the importance of accessing the effects of nisin-biogel sub-MICs at different levels, providing an in vitro basis to understand what happens in vivo throughout the treatment of a DFI, and emphasizes how critical it is to establish the correct dosage of antimicrobials to be applied in clinical practice.

## Data Availability

The data presented in this study are available in the article or Supplementary Materials.

## Supplementary Materials

The following are available online at https://www.mdpi.com/article/10.3390/antibiotics10121501/s1, Table S1: Primers used in RT-qPCR protocols using 7300 Real Time PCR System for accessing virulence gene expression; Table S2: Effects of nisin-biogel at 1/2 MIC, 1/4 MIC and 1/8 MIC and clindamycin at 1/2 MIC on *agrI*, *spA*, *atl*, *clfA*, *coa*, *icaA* and *icaD* mRNA expression for the isolates A 5.2, A 6.3, B 1.1, B 14.2, Z 1.1 and Z 5.2. Results are expressed as n-fold differences in ‘gene under study/*gyrB*’ ratio in the presence of the different conditions described above relative to ‘gene under study/*gyrB*’ ration in the growth control (no antimicrobial). Values are present as mean values ± SD (two repeated different experiments), except for the *clfa/gyrB* fold change for the isolate Z 5.2, since only one assay was performed. Asterisks indicate statistically significant differences between treatments and between treatments and control for each clinical isolate (* = *p* < 0.05; ** = *p* < 0.01; *** = *p* < 0.001, compared with the results of the corresponding control); Table S3: Effects of nisin-biogel at 1/2 MIC, 1/4 MIC and 1/8 MIC and clindamycin at 1/2 MIC on biofilm formation by the isolates A 5.2, A 6.3, B 1.1, B 14.2, Z 1.1 and Z 5.2. Values are present as mean values ± SD (three repeated different experiments). * = *p* < 0.05; ** = *p* < 0.01; *** = *p* < 0.001, compared with the results of the corresponding control; Table S4: Effects of nisin-biogel at 1/2 MIC, 1/4 MIC and 1/8 MIC and clindamycin at 1/2 MIC on coagulase production by the isolates A 5.2, A 6.3, B 1.1, B 14.2, Z 1.1 and Z 5.2 after 24 h of growth under the different conditions tested. Coagulation ability was measured every hour for 4 h of incubation, and after 24 h of incubation. -: no evidence of fibrin formation; 1+: small unorganized clots; 2+: small organized clots; 3+: large organized clots; 4+: entire content of tube coagulates and is not displaced when tube is inverted; Table S5: Effects of nisin-biogel at 1/2 MIC, 1/4 MIC and 1/8 MIC and clindamycin at 1/2 MIC on coagulase production by the isolates A 5.2, A 6.3, B 1.1, B 14.2, Z 1.1 and Z 5.2 after 4 h of growth under the different conditions tested. Coagulation ability was measured every hour for 4 h of incubation, and after 24 h of incubation. -: no evidence of fibrin formation; 1+: small unorganized clots; 2+: small organized clots; 3+: large organized clots; 4+: entire content of tube coagulates and is not displaced when tube is inverted; Table S6: Effects of nisin-biogel at 1/2 MIC, 1/4 MIC and 1/8 MIC and clindamycin at 1/2 MIC on protein A release by *S. aureus* DFI isolates A 5.2, A 6.3, B 1.1, B 14.2, Z 1.1 and Z 5.2 after 18 h of growth under the different conditions tested. The results are the racios of the amount of SpA (pg/mL) in the bacterial supernatants incubated with nisin-biogel or clindamycin to the amount of SpA (pg/mL) in the bacterial supernatants incubated without antimicrobials and are expressed as percentages.
