# Supplementary material for: Influence of Nisin-Biogel at Subinhibitory Concentrations on Virulence Expression in Staphylococcus aureus Isolates from Diabetic Foot Infections
Source: Antibiotics (Basel). 2021 Dec 7;10(12):1501. doi: 10.3390/antibiotics10121501 (PMC8698857; doi:10.3390/antibiotics10121501)
Supplement: Supplementary file 1 [file antibiotics-10-01501-s001.zip › antibiotics-1475743-supplementary.pdf]

Table S1 | Effects of nisin-biogel at 1/2 MIC, 1/4 MIC and 1/8 MIC and clindamycin at 1/2 MIC on *agrI, spA, atl, clfA, coa, icaA* and *icaD* mRNA expression for the isolates A 5.2, A 6.3, B 1.1, B 14.2, Z 1.1 and Z 5.2. Results are expressed as n-fold differences in 'gene under study/*gyrB*' ratio in the presence of the different conditions described above relative to 'gene under study/*gyrB*' ration in the growth control (no antimicrobial). Values are present as mean values ± SD (two repeated different experiments), except for the *clfa*/*gyrB* fold change for the isolate Z 5.2, since only one assay was performed. Asterisks indicate statistically significant differences between treatments and between treatments and control for each clinical isolate (*=p<0.05; **=p<0.01; ***=p<0.001, compared with the results of the corresponding control).

**A 5.2**	***agrI*/*gyrB* fold change**	***spA*/*gyrB* fold change**	***atl*/*gyrB* fold change**	***clfA*/*gyrB* fold change**	***coa*/*gyrB* fold change**	***icaA*/*gyrB* fold change**	***icaD*/*gyrB* fold change**
**Only isolate**	1.00±0.00	1.00±0.00	1.00±0.00	1.00±0.00	1.00±0.00	1.00±0.00	1.00±0.00
**Isolate + 1/2 MIC CLI**	0.04±0.01**	0.34±0.08	2.10±0.15	1.79±0.00**	4.88±3.60	0.33±0.10	0.48±0.12
**Isolate + 1/2 MIC NB**	0.06±0.03*	5.28±1.81	1.11±0.33	0.60±0.24	6.36±0.35	0.79±0.12	1.13±0.03
**Isolate + 1/4 MIC NB**	0.06±0.03*	7.27±3.75	1.09±0.17	0.85±0.37	6.43±1.50	0.83±0.23	1.30±0.13
**Isolate + 1/8 MIC NB**	0.06±0.01**	4.24±0.25*	1.08±0.34	0.45±0.12	3.26±2.69	0.89±0.19	0.89±0.34
**B 14.2**	***agrI*/*gyrB* fold change**	***spA*/*gyrB* fold change**	***atl*/*gyrB* fold change**	***clfA*/*gyrB* fold change**	***coa*/*gyrB* fold change**	***icaA*/*gyrB* fold change**	***icaD*/*gyrB* fold change**
**Only isolate**	1.00±0.00	1.00±0.00	1.00±0.00	1.00±0.00	1.00±0.00	1.00±0.00	1.00±0.00
**Isolate + 1/2 MIC CLI**	0.03±0.02*	0.24±0.04	1.87±0.38	1.28±0.23	2.89±1.15	1.08±0.21	1.10±0.05
**Isolate + 1/2 MIC NB**	0.06±0.02*	0.67±0.17	0.87±0.56	0.31±0.12	2.81±0.23	0.79±0.08	0.77±0.04
**Isolate + 1/4 MIC NB**	0.09±0.02*	0.99±0.31	0.83±0.44	0.29±0.03*	2.05±0.76	0.67±0.12	0.86±0.04
**Isolate + 1/8 MIC NB**	0.05±0.03*	0.61±0.23	0.38±0.11	0.17±0.03*	2.65±0.12*	0.73±0.04	0.97±0.07
**Z 5.2**	***agrI*/*gyrB* fold change**	***spA*/*gyrB* fold change**	***atl*/*gyrB* fold change**	***clfA*/*gyrB* fold change**	***coa*/*gyrB* fold change**	***icaA*/*gyrB* fold change**	***icaD*/*gyrB* fold change**
**Only isolate**	1.00±0.00	1.00±0.00	1.00±0.00	1.00	1.00±0.00	1.00±0.00	1.00±0.00
**Isolate + 1/2 MIC CLI**	0.18±0.09	0.19±0.06*	2.59±1.77	5.54	5.43±4.19	0.40±0.28	0.43±0.27
**Isolate + 1/2 MIC NB**	0.14±0.02*	0.34±0.07	0.69±0.14	0.09	4.67±3.62	1.37±0.29	0.98±0.00
**Isolate + 1/4 MIC NB**	0.15±0.04*	0.36±0.17	0.59±0.09	0.34	8.90±8.12	1.49±0.29	1.06±0.13
**Isolate + 1/8 MIC NB**	0.29±0.1	0.57±0.33	0.55±0.24	1.08	7.83±6.70	1.77±0.53	1.10±0.15
**A 6.3**	***agrI*/*gyrB* fold change**	***spA*/*gyrB* fold change**	***atl*/*gyrB* fold change**	***clfA*/*gyrB* fold change**	***coa*/*gyrB* fold change**	***icaA*/*gyrB* fold change**	***icaD*/*gyrB* fold change**
**Only isolate**	1.00±0.00	1.00±0.00	1.00±0.00	1.00±0.00	1.00±0.00	1.00±0.00	1.00±0.00
**Isolate + 1/2 MIC CLI**	0.24±0.15	1.13±0.68	2.08±0.16	0.76±0.07	12.13±2.45	0.17±0.03	0.23±0.12
**Isolate + 1/2 MIC NB**	0.28±0.07	1.66±1.17	0.75±0.26	0.42±0.07	35.54±15.73	1.10±0.28	1.80±0.18
**Isolate + 1/4 MIC NB**	0.45±0.20	1.39±0.49	0.71±0.14	0.41±0.07	29.38±3.46	1.57±0.43	2.28±0.91
**Isolate + 1/8 MIC NB**	0.41±0.17	2.33±0.75	0.68±0.31	0.52±0.13	19.20±2.71	1.64±0.44	1.80±0.37
**B 1.1**	***agrI*/*gyrB* fold change**	***spA*/*gyrB* fold change**	***atl*/*gyrB* fold change**	***clfA*/*gyrB* fold change**	***coa*/*gyrB* fold change**	***icaA*/*gyrB* fold change**	***icaD*/*gyrB* fold change**
**Only isolate**	1.00±0.00	1.00±0.00	1.00±0.00	1.00±0.00	1.00±0.00	1.00±0.00	1.00±0.00
**Isolate + 1/2 MIC CLI**	0.20±0.16	0.29±0.11	0.35±0.26	1.32±0.85	7.54±0.07**	1.55±0.49	1.33±0.02*
**Isolate + 1/2 MIC NB**	0.10±0.05*	0.46±0.10	0.14±0.10	0.29±0.21	9.94±3.05	0.73±0.09	1.04±0.06
**Isolate + 1/4 MIC NB**	0.13±0.04*	0.71±0.42	0.11±0.07	0.28±0.15	7.73±1.59	0.91±0.07	1.14±0.01*

**Isolate + 1/8 MIC NB**	0.20±0.03*	0.98±0.47	0.14±0.10	0.33±0.11	8.09±0.48*	1.40±0.30	1.60±0.45
**Z 1.1**	***agrI*/*gyrB* fold change**	***spA*/*gyrB* fold change**	***atl*/*gyrB* fold change**	***clfA*/*gyrB* fold change**	***coa*/*gyrB* fold change**	***icaA*/*gyrB* fold change**	***icaD*/*gyrB* fold change**
**Only isolate**	1.00±0.00	1.00±0.00	1.00±0.00	1.00±0.00	1.00±0.00	1.00±0.00	1.00±0.00
**Isolate + 1/2 MIC CLI**	0.07±0.04*	0.19±0.08	0.57±0.01*	0.82±0.20	1.46±0.02*	0.64±0.61	1.19±0.09
**Isolate + 1/2 MIC NB**	0.14±0.03	2.01±0.54	0.23±0.05*	0.27±0.04*	2.71±0.16	0.98±0.22	0.94±0.05
**Isolate + 1/4 MIC NB**	0.13±0.09	1.10±0.19	0.18±0.06*	0.24±0.11	2.73±0.57	1.17±0.48	0.99±0.04
**Isolate + 1/8 MIC NB**	0.15±0.04	1.03±0.19	0.28±0.09	0.27±0.08	2.43±0.05*	1.52±0.19	1.99±0.02**

NB: nisin-biogel; CLI: clindamycin; MIC: minimum inhibitory concentration. *agrI*: accessory gene regulator I; *spA:* gene encoding staphylococcal protein A; *atl:* gene encoding autolysin; *clfA:* gene encoding clumping factor A; *coa:* gene encoding coagulase; *icaA:* gene encoding intracellular adhesin A; *icaD:* gene encoding intracellular adhesin D; *gyrB:* gene encoding gyrase B.

Table S2 | Effects of nisin-biogel at 1/2 MIC, 1/4 MIC and 1/8 MIC and clindamycin at 1/2 MIC on biofilm formation by the isolates A 5.2, A 6.3, B 1.1, B 14.2, Z 1.1 and Z 5.2. Values are present as mean values ± SD (three repeated different experiments). *=p<0.05; **=p<0.01; ***=p<0.001, compared with the results of the corresponding control.

	**Only isolate**	**Isolate + 1/2 MIC CLI**	**Isolate + 1/2 MIC NB**	**Isolate + 1/4 MIC NB**	**Isolate + 1/8 MIC NB**
**A 5.2**	0.49±0.08	0.49±0.15	0.28±0.04	0.34±0.01	0.46±0.10
**B 14.2**	0.21±0.06	0.25±0.03	0.37±0.09	0.39±0.11	0.54±0.13
**Z 5.2**	0.25±0.04	0.27±0.05	0.21±0.04	0.33±0.05**	0.44±0.16
**A 6.3**	0.36±0.10	0.31±0.03	0.40±0.06	0.36±0.13	0.44±0.18
**B 1.1**	0.23±0.02	0.24±0.02	0.26±0.12	0.38±0.06	0.50±0.17
**Z 1.1**	0.25±0.02	0.37±0.08	0.27±0.04	0.30±0.12	0.35±0.11

NB: nisin-biogel; CLI: clindamycin; MIC: minimum inhibitory concentration.

Table S3 | Effects of nisin-biogel at 1/2 MIC, 1/4 MIC and 1/8 MIC and clindamycin at 1/2 MIC on coagulase production by the isolates A 5.2, A 6.3, B 1.1, B 14.2, Z 1.1 and Z 5.2 after 4h of growth under the different conditions tested. Coagulation ability was measured every hour for 4h of incubation, and after 24h of incubation. -: no evidence of fibrin formation; 1+: small unorganized clots; 2+: small organized clots; 3+: large organized clots; 4+: entire content of tube coagulates and is not displaced when tube is inverted.

	**Only isolate**	**Isolate + 1/2 MIC CLI**	**Isolate + 1/2 MIC NB**	**Isolate + 1/4 MIC NB**	**Isolate + 1/8 MIC NB**
**A 5.2 E1**	1h – -<br>2h – 3+<br>3h – 3+<br>4h – 3+<br>24h – 4+	1h – -<br>2h – -<br>3h – -<br>4h – -<br>24h – 4+	1h – -<br>2h – 2+<br>3h – 3+<br>4h – 3+<br>24h – 3+	1h – 1+<br>2h – 4+<br>3h – 4+<br>4h – 4+<br>24h – 4+	1h – 2+<br>2h – 4+<br>3h – 4+<br>4h – 4+<br>24h – 4+
**A 5.2 E2**	1h – -<br>2h – 3+<br>3h – 3+<br>4h – 4+<br>24h – 4+	1h – -<br>2h – -<br>3h – -<br>4h – -<br>24h – 4+	1h – -<br>2h – -<br>3h – 2+<br>4h – 2+<br>24h – 4+	1h – -<br>2h – 3+<br>3h – 3+<br>4h – 3+<br>24h – 4+	1h – -<br>2h – 3+<br>3h – 3+<br>4h – 3+<br>24h – 4+
**B 14.2 E1**	1h – -<br>2h – 1+<br>3h – 3+<br>4h – 4+<br>24h – 4+	1h – -<br>2h – -<br>3h – -<br>4h – -<br>24h – 4+	1h – -<br>2h – -<br>3h – -<br>4h – 1+<br>24h – 3+	1h – 1+<br>2h – 3+<br>3h – 3+<br>4h – 3+<br>24h – 4+	1h – 2+<br>2h – 3+<br>3h – 3+<br>4h – 3+<br>24h – 4+
**B 14.2 E2**	1h – -<br>2h – 2+<br>3h – 2+<br>4h – 2+<br>24h – 3+	1h – -<br>2h – -<br>3h – -<br>4h – 1+<br>24h – 4+	1h – -<br>2h – -<br>3h – -<br>4h – -<br>24h – 3+	1h – -<br>2h – -<br>3h – -<br>4h – 2+<br>24h – 3+	1h – -<br>2h – 3+<br>3h – 3+<br>4h – 3+<br>24h – 3+
**Z 5.2 E1**	1h – 2+<br>2h – 4+<br>3h – 4+<br>4h – 4+<br>24h – 4+	1h – -<br>2h – -<br>3h – -<br>4h – -<br>24h – 4+	1h – -<br>2h – -<br>3h – -<br>4h – -<br>24h – 4+	1h – -<br>2h – -<br>3h – -<br>4h – 1+<br>24h – 4+	1h – -<br>2h – 4+<br>3h – 4+<br>4h – 4+<br>24h – 4+
**Z 5.2 E2**	1h – 2+<br>2h – 3+<br>3h – 3+<br>4h – 3+<br>24h – 3+	1h – -<br>2h – -<br>3h – -<br>4h – -<br>24h – 3+	1h – -<br>2h – -<br>3h – -<br>4h – -<br>24h – 3+	1h – -<br>2h – -<br>3h – -<br>4h – -<br>24h – 3+	1h – -<br>2h – 3+<br>3h – 3+<br>4h – 3+<br>24h – 3+
**A 6.3 E1**	1h – -<br>2h – -<br>3h – 4+<br>4h – 4+<br>24h – 4+	1h – -<br>2h – -<br>3h – -<br>4h – -<br>24h – 4+	1h – -<br>2h – -<br>3h – -<br>4h – 2+<br>24h – 4+	1h – -<br>2h – 2+<br>3h – 3+<br>4h – 3+<br>24h – 4+	1h – 1+<br>2h – 4+<br>3h – 4+<br>4h – 4+<br>24h – 4+
**A 6.3 E2**	1h – -<br>2h – 1+<br>3h – 3+<br>4h – 3+<br>24h – 4+	1h – -<br>2h – -<br>3h – -<br>4h – -<br>24h – 4+	1h – -<br>2h – -<br>3h – -<br>4h – -<br>24h – 3+	1h – -<br>2h – -<br>3h – 1+<br>4h – 2+<br>24h – 4+	1h – -<br>2h – 1+<br>3h – 3+<br>4h – 3+<br>24h – 4+
**B 1.1 E1**	1h – 2+<br>2h – 3+<br>3h – 3+<br>4h – 3+<br>24h – 4+	1h – -<br>2h – -<br>3h – -<br>4h – -<br>24h – 4+	1h – 2+<br>2h – 3+<br>3h – 3+<br>4h – 3+<br>24h – 4+	1h – 3+<br>2h – 3+<br>3h – 3+<br>4h – 3+<br>24h – 4+	1h – 3+<br>2h – 3+<br>3h – 3+<br>4h – 3+<br>24h – 4+
**B 1.1 E2**	1h – -<br>2h – 3+<br>3h – 4+<br>4h – 4+<br>24h – 4+	1h – -<br>2h – -<br>3h – -<br>4h – -<br>24h – 4+	1h – -<br>2h – -<br>3h – -<br>4h – 1+<br>24h – 4+	1h – -<br>2h – 3+<br>3h – 3+<br>4h – 3+<br>24h – 4+	1h – 1+<br>2h – 3+<br>3h – 3+<br>4h – 3+<br>24h – 4+

**Z 1.1 E1**	1h – 3+<br>2h – 3+<br>3h – 3+<br>4h – 3+<br>24h – 4+	1h – -<br>2h – -<br>3h – -<br>4h – -<br>24h – 4+	1h – 2+<br>2h – 3+<br>3h – 3+<br>4h – 3+<br>24h – 4+	1h – 3+<br>2h – 3+<br>3h – 3+<br>4h – 3+<br>24h – 4+	1h – 3+<br>2h – 3+<br>3h – 3+<br>4h – 3+<br>24h – 4+
**Z 1.1 E2**	1h – 3+<br>2h – 3+<br>3h – 3+<br>4h – 3+<br>24h – 4+	1h – -<br>2h – -<br>3h – -<br>4h – -<br>24h – 4+	1h – -<br>2h – -<br>3h – -<br>4h – 1+<br>24h – 4+	1h – 1+<br>2h – 3+<br>3h – 3+<br>4h – 3+<br>24h – 4+	1h – 3+<br>2h – 3+<br>3h – 3+<br>4h – 3+<br>24h – 4+

NB: nisin-biogel; CLI: clindamycin; MIC: minimum inhibitory concentration.

Table S4 | Effects of nisin-biogel at 1/2 MIC, 1/4 MIC and 1/8 MIC and clindamycin at 1/2 MIC on coagulase production by the isolates A 5.2, A 6.3, B 1.1, B 14.2, Z 1.1 and Z 5.2 after 24h of growth under the different conditions tested. Coagulation ability was measured every hour for 4h of incubation, and after 24h of incubation. -: no evidence of fibrin formation; 1+: small unorganized clots; 2+: small organized clots; 3+: large organized clots; 4+: entire content of tube coagulates and is not displaced when tube is inverted.

	**Only isolate**	**Isolate + 1/2 MIC CLI**	**Isolate + 1/2 MIC NB**	**Isolate + 1/4 MIC NB**	**Isolate + 1/8 MIC NB**
**A 5.2 E1**	1h – 4+<br>2h – 4+<br>3h – 4+<br>4h – 4+<br>24h – 4+	1h – -<br>2h – -<br>3h – -<br>4h – -<br>24h – -	1h – 2+<br>2h – 2+<br>3h – 3+<br>4h – 3+<br>24h – 3+	1h – 4+<br>2h – 4+<br>3h – 4+<br>4h – 4+<br>24h – 4+	1h – 4+<br>2h – 4+<br>3h – 4+<br>4h – 4+<br>24h – 4+
**A 5.2 E2**	1h – 2+<br>2h – 3+<br>3h – 3+<br>4h – 3+<br>24h – 3+	1h – -<br>2h – -<br>3h – -<br>4h – -<br>24h – -	1h – 2+<br>2h – 3+<br>3h – 3+<br>4h – 3+<br>24h – 3+	1h – 3+<br>2h – 3+<br>3h – 3+<br>4h – 3+<br>24h – 3+	1h – 2+<br>2h – 3+<br>3h – 3+<br>4h – 3+<br>24h – 3+
**B 14.2 E1**	1h – 4+<br>2h – 4+<br>3h – 4+<br>4h – 4+<br>24h – 4+	1h – 4+<br>2h – 4+<br>3h – 4+<br>4h – 4+<br>24h – 4+	1h – 2+<br>2h – 3+<br>3h – 3+<br>4h – 3+<br>24h – 3+	1h – 4+<br>2h – 4+<br>3h – 4+<br>4h – 4+<br>24h – 4+	1h – 4+<br>2h – 4+<br>3h – 4+<br>4h – 4+<br>24h – 4+
**B 14.2 E2**	1h – 2+<br>2h – 3+<br>3h – 3+<br>4h – 3+<br>24h – 3+	1h – 3+<br>2h – 3+<br>3h – 3+<br>4h – 3+<br>24h – 3+	1h – 2+<br>2h – 3+<br>3h – 3+<br>4h – 3+<br>24h – 3+	1h – 2+<br>2h – 3+<br>3h – 3+<br>4h – 3+<br>24h – 3+	1h – 2+<br>2h – 3+<br>3h – 3+<br>4h – 3+<br>24h – 3+
**Z 5.2 E1**	1h – 4+<br>2h – 4+<br>3h – 4+<br>4h – 4+<br>24h – 4+	1h – -<br>2h – -<br>3h – -<br>4h – -<br>24h – -	1h – 4+<br>2h – 4+<br>3h – 4+<br>4h – 4+<br>24h – 2+	1h – 4+<br>2h – 4+<br>3h – 4+<br>4h – 4+<br>24h – 3+	1h – 3+<br>2h – 3+<br>3h – 3+<br>4h – 3+<br>24h – 3+
**Z 5.2 E2**	1h – 2+<br>2h – 3+<br>3h – 3+<br>4h – 3+<br>24h – 3+	1h – -<br>2h – -<br>3h – -<br>4h – -<br>24h – -	1h – 2+<br>2h – 3+<br>3h – 3+<br>4h – 3+<br>24h – 3+	1h – 2+<br>2h – 3+<br>3h – 3+<br>4h – 3+<br>24h – 3+	1h – 2+<br>2h – 3+<br>3h – 3+<br>4h – 3+<br>24h – 3+
**A 6.3 E1**	1h – 2+<br>2h – 3+<br>3h – 3+<br>4h – 3+<br>24h – 4+	1h – -<br>2h – -<br>3h – -<br>4h – -<br>24h – -	1h – 2+<br>2h – 3+<br>3h – 3+<br>4h – 3+<br>24h – 4+	1h – 2+<br>2h – 3+<br>3h – 3+<br>4h – 3+<br>24h – 4+	1h – 2+<br>2h – 3+<br>3h – 3+<br>4h – 3+<br>24h – 3+
**A 6.3 E2**	1h – 3+<br>2h – 3+<br>3h – 3+<br>4h – 3+<br>24h – 4+	1h – -<br>2h – -<br>3h – -<br>4h – -<br>24h – -	1h – 3+<br>2h – 3+<br>3h – 3+<br>4h – 3+<br>24h – 4+	1h – 3+<br>2h – 3+<br>3h – 3+<br>4h – 3+<br>24h – 4+	1h – 3+<br>2h – 3+<br>3h – 3+<br>4h – 3+<br>24h – 4+
**B 1.1 E1**	1h – 2+<br>2h – 3+<br>3h – 3+<br>4h – 3+<br>24h – 3+	1h – 2+<br>2h – 4+<br>3h – 4+<br>4h – 4+<br>24h – 4+	1h – 4+<br>2h – 4+<br>3h – 4+<br>4h – 4+<br>24h – 4+	1h – 2+<br>2h – 3+<br>3h – 3+<br>4h – 3+<br>24h – 3+	1h – 2+<br>2h – 3+<br>3h – 3+<br>4h – 3+<br>24h – 3+
**B 1.1 E2**	1h – 3+<br>2h – 3+<br>3h – 3+<br>4h – 3+<br>24h – 4+	1h – 3+<br>2h – 3+<br>3h – 3+<br>4h – 3+<br>24h – 4+	1h – 3+<br>2h – 3+<br>3h – 3+<br>4h – 3+<br>24h – 4+	1h – 3+<br>2h – 3+<br>3h – 3+<br>4h – 3+<br>24h – 4+	1h – 3+<br>2h – 3+<br>3h – 3+<br>4h – 3+<br>24h – 4+
**Z 1.1 E1**	1h – 2+<br>2h – 3+<br>3h – 3+<br>4h – 3+	1h – -<br>2h – 3+<br>3h – 3+<br>4h – 3+	1h – 3+<br>2h – 4+<br>3h – 4+<br>4h – 4+	1h – 4+<br>2h – 4+<br>3h – 4+<br>4h – 4+	1h – 4+<br>2h – 4+<br>3h – 4+<br>4h – 4+

	24h – 3+	24h – 3+	24h – 4+	24h – 4+	24h – 4+
**Z 1.1 E2**	1h – 3+<br>2h – 3+<br>3h – 3+<br>4h – 3+<br>24h – 3+	1h – 3+<br>2h – 3+<br>3h – 3+<br>4h – 3+<br>24h – 4+	1h – 3+<br>2h – 3+<br>3h – 3+<br>4h – 3+<br>24h – 3+	1h – 3+<br>2h – 3+<br>3h – 3+<br>4h – 3+<br>24h – 3+	1h – 3+<br>2h – 3+<br>3h – 3+<br>4h – 3+<br>24h – 3+

NB: nisin-biogel; CLI: clindamycin; MIC: minimum inhibitory concentration.

Table S5 | Effects of nisin-biogel at 1/2 MIC, 1/4 MIC and 1/8 MIC and clindamycin at 1/2 MIC on protein A release by *S. aureus* DFI isolates A 5.2, A 6.3, B 1.1, B 14.2, Z 1.1 and Z 5.2 after 18h of growth under the different conditions tested. The results are the racios of the amount of SpA (pg/mL) in the bacterial supernatants incubated with nisin-biogel or clindamycin to the amount of SpA (pg/mL) in the bacterial supernatants incubated without antimicrobials and are expressed as percentages.

	**A 5.2**	**A 6.3**	**B 1.1**	**B 14.2**	**Z 1.1**	**Z 5.2**
**No antimicrobial**	100%	100%	100%	100%	100%	100%
**1/2 MIC CLI**	49%	150%	55%	79%	42%	113%
**1/2 MIC NB**	74%	95%	61%	46%	56%	109%
**1/4 MIC NB**	77%	98%	111%	69%	68%	96%
**1/8 MIC NB**	105%	91%	130%	66%	62%	86%

NB: nisin-biogel; CLI: clindamycin

Table S6 | Primers used in RT-qPCR protocols using 7300 Real Time PCR System for accessing virulence gene expression.

Primer	Sequence (5′-3′)	Reference
***gyrB* F**	GGTGGCGACTTTGATCTAGC	Otto et al., 2013 [41]
***gyrB* R**	TTATACAACGGTGGCTGTGC	Otto et al., 2013 [41]
***agrI* F**	CCAGCTATAATTAGTGGTATTAAGTACAGTAAACT	Francois et al., 2006 [50]
***agrI* R**	AGGACGCGCTATCAAACATTTT	Francois et al., 2006 [50]
***spA* F**	TATGCCTAACTTAAATGCTG	Otto et al., 2013 [41]
***spA* R**	TTGGAGCTTGAGAGTCATTA	Otto et al., 2013 [41]
***atl* F**	ACCAAGATCAGTTGCTGCAA	This study
***atl* R**	CGGTATGCGTATTTAGGGAAGT	This study
***clfA* F**	ACCCAGGTTCAGATTCTGGCAGCG	Atshan et al., 2013 [51]
***clfA* R**	TCGCTGAGTCGGAATCGCTTGCT	Atshan et al., 2013 [51]
***coa* F**	GTAGATTGGGCAATTACATTTTGGAGG	Kearns et al., 1999 [52]
***coa* R**	CGCATCAGCTTTGTTATCCCATGT	Kearns et al., 1999 [52]
***icaA* F**	GAGGTAAAGCCAACGCACTC	Atshan et al., 2013 [51]
***icaA* R**	CCTGTTAACCGCACCAAGTTT	Atshan et al., 2013 [51]
***icaD* F**	ACCCAACGCTAAAATCATCG	Atshan et al., 2013 [51]
***icaD* R**	GCGAAAATGCCCATAGTTTC	Atshan et al., 2013 [51]

gyrB: gene encoding gyrase B; *agrI:* accessory gene regulator I; *spA:* gene encoding protein A; *atl:* gene encoding autolysin; *clfA:* gene encoding clumping factor A; *coa:* gene encoding coagulase; *icaA:* gene encoding intracellular adhesin A; *icaD:* gene encoding intracellular adhesin D.
